# Diplotype-Guided Differences in Alcohol Withdrawal Severity Under Diazepam Therapy

**DOI:** 10.1192/j.eurpsy.2026.10485

**Published:** 2026-07-31

**Authors:** S. Özkan-Kotiloğlu, D. Kaya-Akyüzlü, M. Danışman, M. A. Yıldırım, I. Özgür-İlhan, S. Süzen

**Affiliations:** 1 Department of Molecular Biology and Genetics, Kırşehir Ahi Evran University, Kırşehir; 2 Institute of Forensic Sciences, Ankara University; 3 AMATEM Clinic, Ankara Training and Research Hospital; 4 Department of Mental Health and Diseases; 5 Department of Pharmaceutical Toxicology, Ankara University, Ankara, Türkiye

## Abstract

**Introduction:**

Benzodiazepines are the first-line treatment for Alcohol Withdrawal Syndrome (AWS), with diazepam (DZP) favoured for its long half-life and effective symptom control. DZP is primarily metabolised by CYP2C19 enzyme, whose activity can vary significantly due to genetic polymorphisms, leading to differences in treatment response among individuals.

**Objectives:**

This study aimed to assess the association between *CYP2C19* variants and alcohol withdrawal severity in patients undergoing DZP treatment.

**Methods:**

A total of 97 male patients diagnosed with AWS and receiving oral DZP therapy were recruited. Genotyping for CYP2C19*2 and CYP2C19*17 polymorphisms was performed using PCR-RFLP. The Clinical Institute Withdrawal Assessment for Alcohol Scale-Revised (CIWA-Ar) was used to evaluate withdrawal severity after 10-days oral DZP treatment. The impact of different *CYP2C19* genotypes and metaboliser phenotypes (EMs: extensive metabolisers: *1/*1 diplotype; IMs: Intermediate metabolisers: *1/*2 or *2/*17 diplotype; PMs: poor metabolisers: *2/*2 diploype; RMs: rapid metabolisers: *1/*17 diplotype; UMs: ultra rapid metabolisers: *17/*17 diplotype) on CIWA-Ar scores was statistically analysed.

**Results:**

A significant association was observed between CYP2C19*17 genotypes and CIWA-Ar scores in the co-dominant model (CC: median 5.00 [IQR: 2.00 – 10.00]; CT: 5.00 [3.50 – 9.50]; TT: 1.00 [1.00 – 3.50]; *p*=0.043). Post-hoc analysis under the C-dominant model revealed a statistically significant difference between TT and CC+CT groups (*p*=0.013), whereas the T-dominant model showed no significant difference (*p*=0.529). No significant association was found between CYP2C19*2 genotypes and withdrawal scores (*p*=0.941). Notably, UMs exhibited significantly lower alcohol withdrawal scores (median 1.00 [IQR: 1.00 – 3.50]) compared to EMs, who had a median score of 6.00 [IQR: 2.25 – 9.75] (*p*=0.012).

**Conclusions:**

This study demonstrates that CYP2C19*17 polymorphism significantly influences DZP’s effectiveness in managing AWS. Individuals with UM profile, experienced milder withdrawal symptoms compared to EMs. These findings suggest that pharmacogenetic profiling of *CYP2C19* could give personalised DZP therapy in AWS, potentially improving treatment outcomes.

*This study was supported by Scientific and Technological Research Council of Turkey (TUBITAK) under the Grant Number 121C441. The authors thank to TUBITAK for their supports.*

**Disclosure of Interest:**

None Declared

